# Modified putty index matrix technique with mylar strip and a new classification for selecting the type of matrix in anterior proximal/incisal composite restorations

**DOI:** 10.1002/ccr3.1006

**Published:** 2017-06-01

**Authors:** I. Anand Sherwood, Mensudar Rathakrishnan, Kamatchi Subramanian Savadamaoorthi, Puridi Bhargavi, Vasanthan Vignesh Kumar

**Affiliations:** ^1^ Department of Conservative Dentistry and EndodonticsCSI College of Dental Sciences and ResearchMaduraiTamil NaduIndia; ^2^ Department of Conservative Dentistry and EndodonticsSree Balaji Dental College and HospitalChennaiTamil NaduIndia; ^3^ Department of OrthodonticsCSI College of Dental Sciences and ResearchMaduraiTamil NaduIndia

**Keywords:** Bonding, class IV composites, composites, contact and contour, esthetics, mylar strip, putty index

## Abstract

Matrix technique described in this article combines the advantages of both flexible and rigid matrix in anterior composite restorations. Using mylar strip provide advantages, of one utilizing the mylar strip for contouring the labial aspect of restoration thereby, and overcomes the problem in adapting the teflon tape around the tooth.

## Introduction

Composites are one of the most used materials for rehabilitation of anterior teeth defects, so an extensive knowledge on use of this material is essential for a clinician 1. One of the main challenges in anterior composite restorations is in establishing and reproducing the proper contour and contact form. Matrix application is a critical step in achieving this objective in anterior composite restorations.

**Figure 1:**
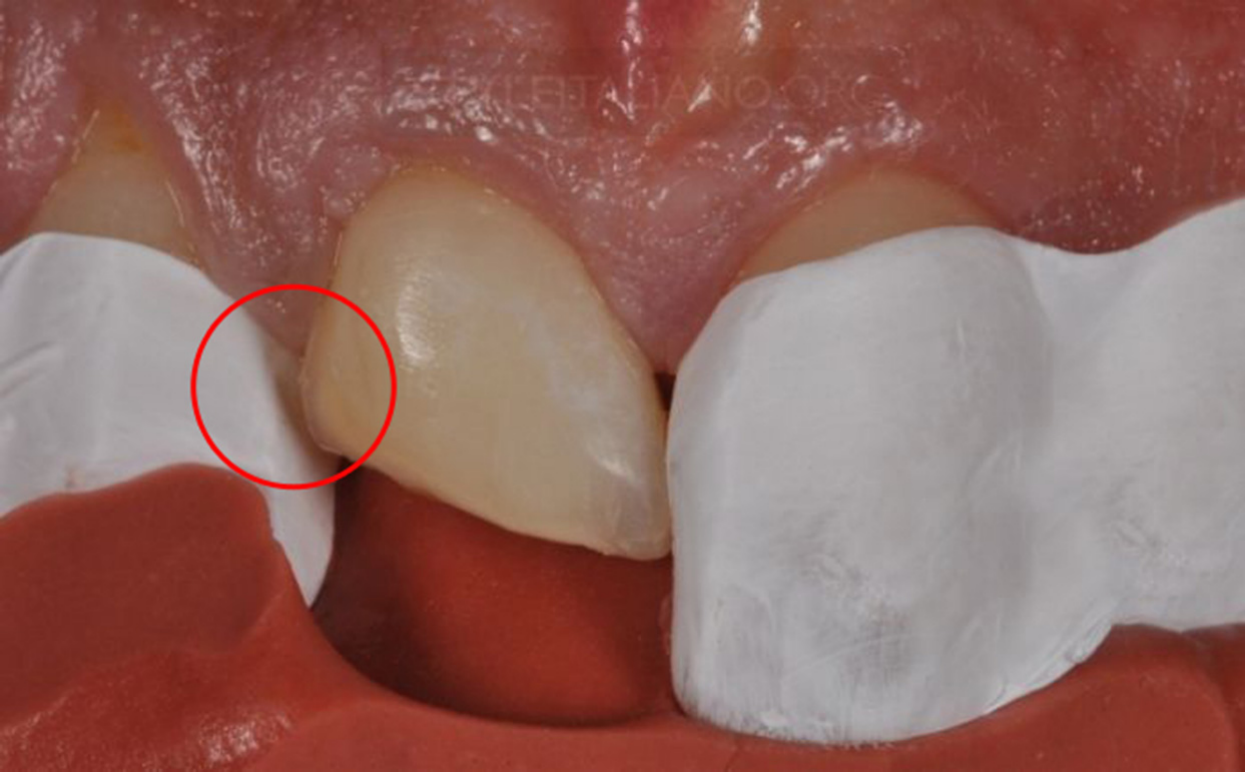
Showing gap between teflon tape and tooth structure.

Various matrices are available for anterior composite restorations; they can be broadly classified into flexible and rigid types 2. Flexible matrices are mylar or cellophane strips, soft splint templates, and rigid matrices are compound supported matrix, putty index matrix. The more popular and widely used matrices are the mylar strips and the putty index. Advantages with mylar strip are that it is easy to apply, no need for fabrication of impression or mock build up; disadvantages are its flexibility which can lead to improper contour and contact establishment especially in large defect restorations, impossible to obtain exact correct contour in the palatal aspect of restorations. Advantages with putty index matrix are that exact palatal contour and form can be obtained, since it is a rigid matrix exact contour can be obtained even in large defects and also can be used predictably to restore multiple defects; disadvantages are that a mock build up of the defect has to be made, may require second appointment, composite build up can adhere to the adjacent tooth especially if the adjacent tooth is not isolated. Many authors have described technique for placement of composite restorations for anterior teeth with putty index alone or putty index combined with flexible matrix (Polytetrafluoroethylene [PTFE] Teflon tape) 3, 4, 5, 6, 7, 8, 9, 10. The major concerns encountered with use of PTFE Teflon tape or plumber's tape are its difficulty to manipulate around the teeth, many times it gets stuck in the contact area and difficulty in removal after the restorations has been completed, and because of the inability of the teflon tape to be pulled over the labial aspect of tooth structure while polymerizing the composite proper labial contour of restoration is not predictably achieved which can lead to gingival overhang of composites especially when the defect is extending gingival to the height of contour or contact area (Fig. 1). So instead of teflon tape, in this article mylar strip is being advocated.

This article discusses about a modification with application of putty index as matrix along with mylar strip for anterior proximal/incisal composite restorations and a new classification to guide in selection of putty index matrix for anterior composite restoration.

## Clinical Technique Description

### Modified putty index matrix technique with mylar strip

After the defect to be restored in anterior teeth is build up using either wax or mock composite build up in the model of patient's teeth (indirect technique) or directly from the patient mouth (Direct technique) a putty index of the anterior teeth is made. The index is made in conventional method using either addition or condensation silicone putty material but addition of silicone material is preferred choice. The index is required to cover whole palatal surface of the tooth to be restored and extend at least two teeth on either side of the tooth to be restored and cover just the incisal tips of the teeth without extending over the labial side of tooth surface.

**Figure 2:**
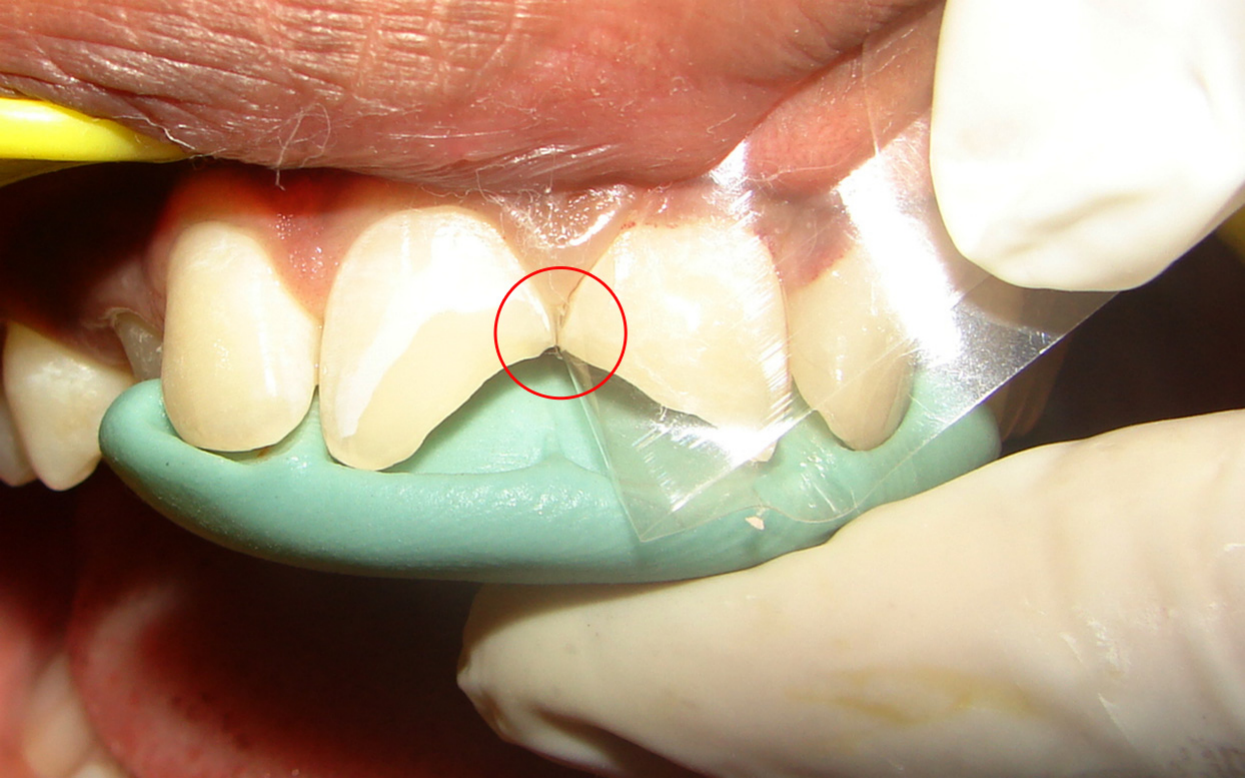
Mylar strip and putty index in patients tooth. No gap present between tooth structure and mylar strip.

**Figure 3:**
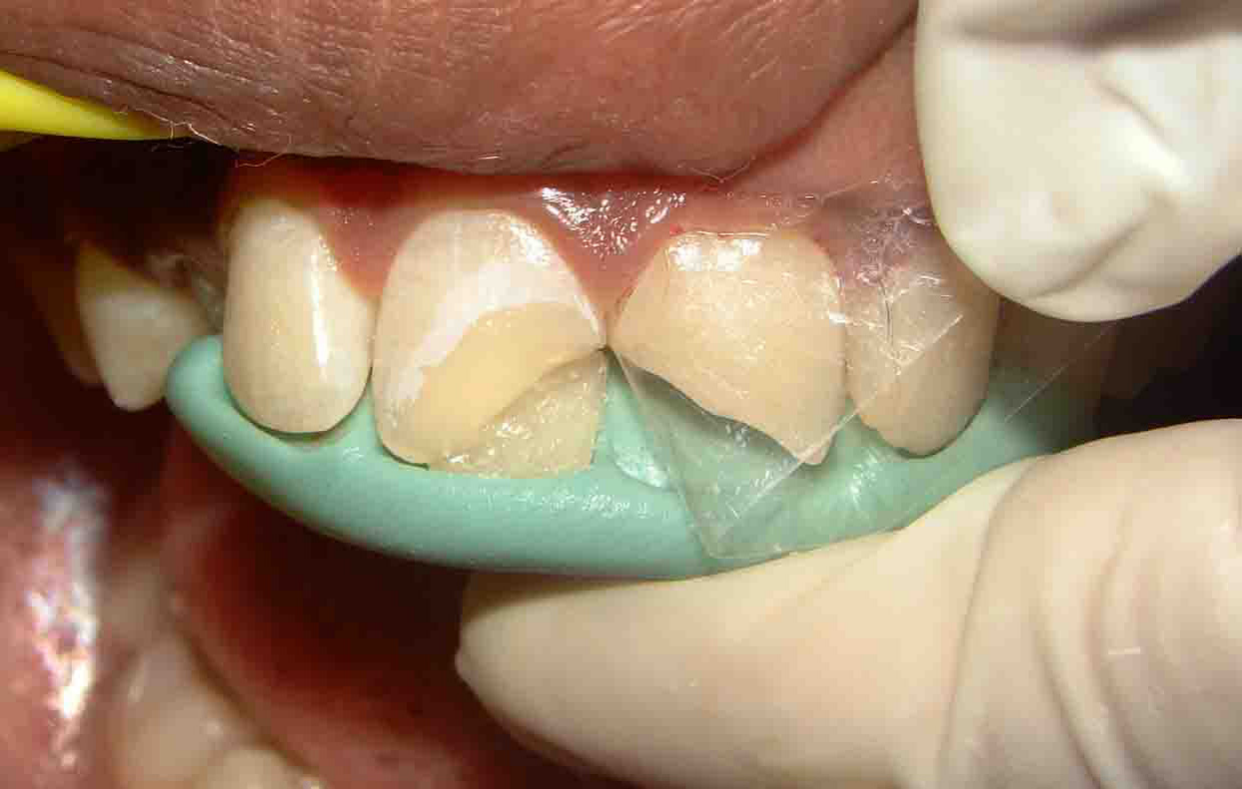
Palatal shelf build up done with mylar strip and index in place.

**Figure 4:**
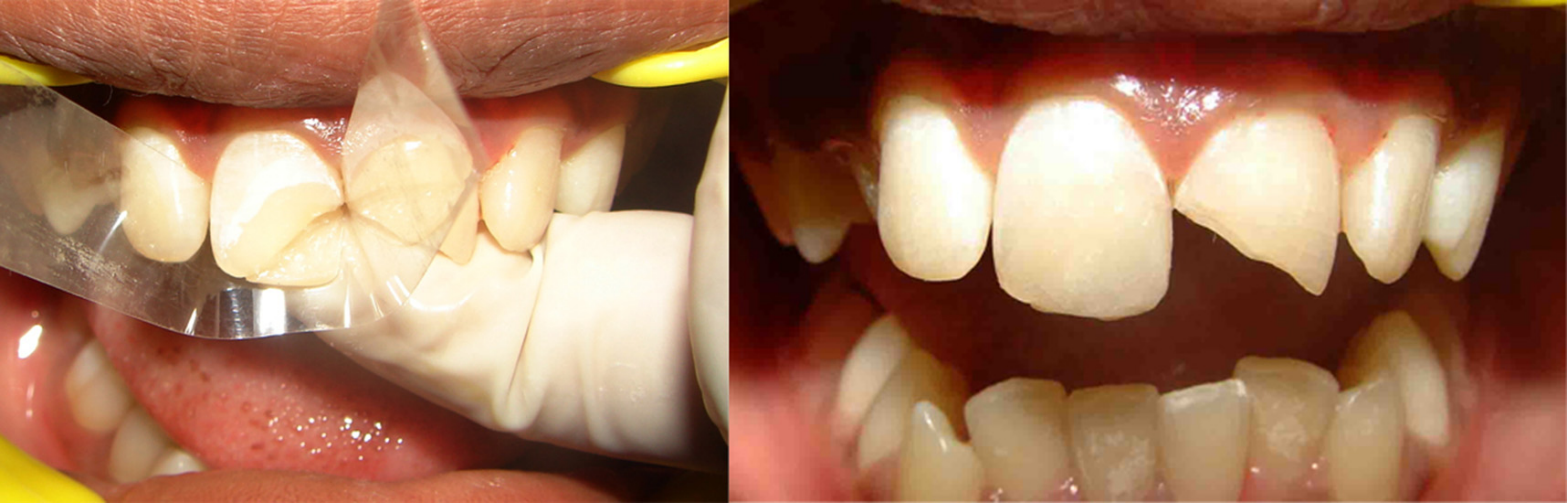
Labial aspect build up with mylar strip being used to pull over the labial surface.

The fabricated the putty index is placed in the patients’ teeth and checked for fit and extensions. After acid etching and bonding agent application to the tooth to be restored is completed, the putty index is placed on the palatal side for composite placement. A modification at this stage from the conventional method is placement of mylar strip on the adjacent tooth to prevent composite material adhering to the adjacent tooth surface (Fig. 2). Once the mylar strip has been placed, the putty index is placed on palatal surface of teeth followed by composite placement. Composite is placed with putty index and mylar strip in place, and a palatal wall/shelf is created and is not extended labially beyond the middle of the contact point or contact area (Fig. 3). Once the palatal wall/shelf of composite is created to the necessary dimensions, the putty index is removed and the labial part of the restoration is performed with help of application of only mylar strip being pulled over to labial surface to get the desired labial contour (Fig. 4).

Advantages of this technique of combining both rigid and flexible matrices for restoring defects are,

1. Rigid matrix when used for restoring the palatal surface gives desired contour and length/extension of incisal edge, which in turn can guide and support the labial surface composite build up. And versatility of this technique it can also aid in moisture control for palatal surface.
2. Flexible matrix mylar strip placement prevents composite from adhering onto adjacent tooth, and when using in labial surface build up helps in achieving the desired esthetic anatomic contour and excellent labial surface finish.
3. Can be used even in difficult cases like, multiple teeth restorations, crowded teeth, and extensive defect restorations.

Limitations of this technique will be, restorations might require two appointments for patient and training in placing both mylar strip and index together in initial stages of usage. Also this technique will necessitate the practice of four handed dentistry, as operator will require to manipulate and place both mylar strip and putty index together at same instance.

## Case Diagnosis

### Newer classification for anterior proximoincisal preparation

Newer classification was needed as many systems of matrices and techniques for anterior proximoincisal composite restoration placement are available, and relying on a system of classification of only knowing the site of defect like G. V. Black's or G. J. Mount classifications will not be sufficient for selecting one method of matricing over other 1, 2, 3, 4, 5, 6, 7, 8, 9, 10. So the newer classification was drafted to incorporate these needs. And the goal of this classification is to guide the clinician in selecting appropriate matrix system in restoration of anterior teeth with composite restoration.

This newer classification is based on need that a stiffer and a well supporting putty index matrix will be required when restoring proximal defects which are greater in dimensions to satisfactorily re‐establish adequate contour and contact form, than compared to defects which are smaller where flexible mylar strip will suffice. And also stiffer putty index will be required for defects which are going extend from labial to palatal surfaces (through and through defects).

**Figure 5:**
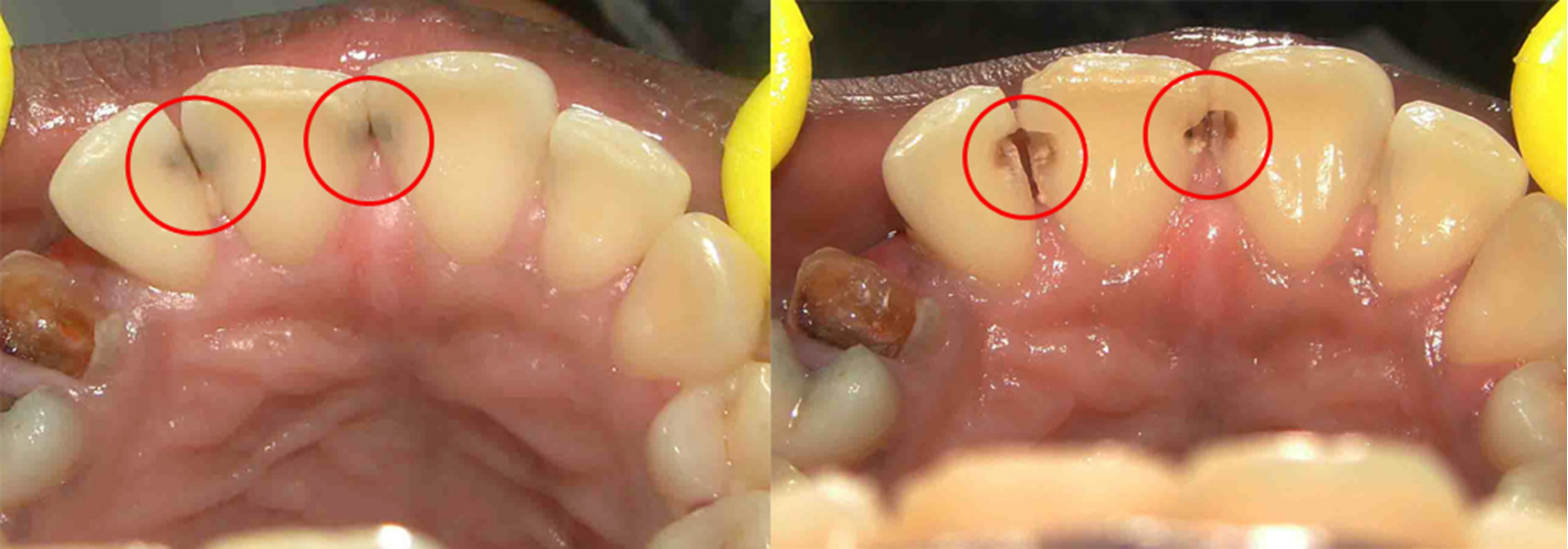
Defects present only on palatal surface. Type 1.

**Figure 6:**
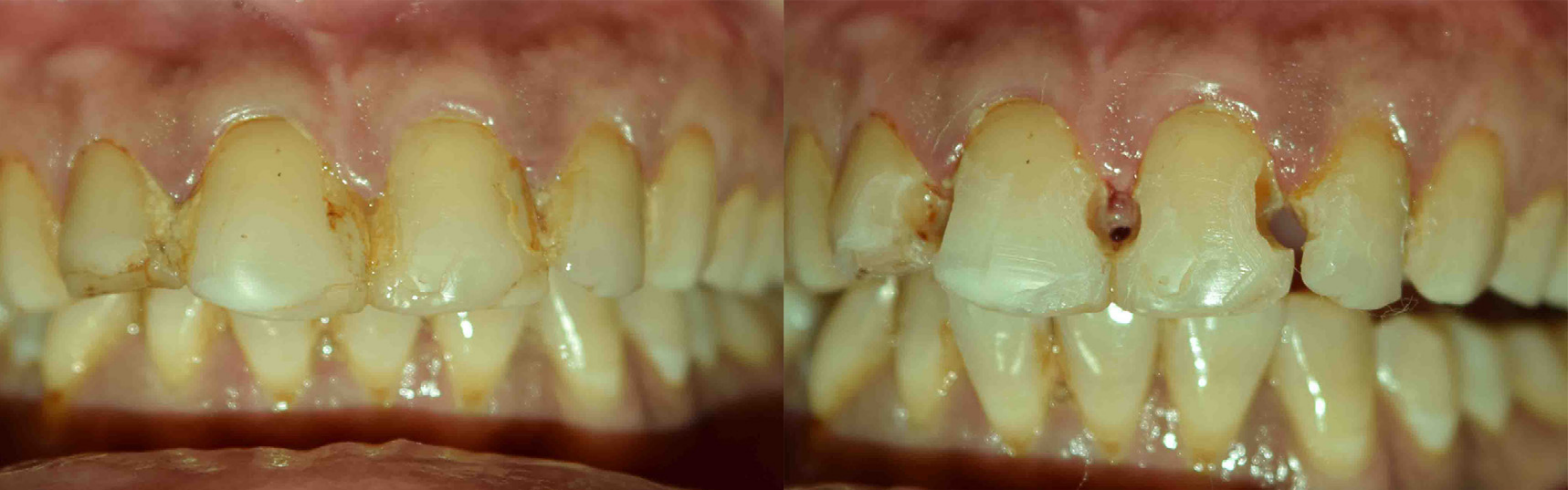
Defects less than 2/3rd the inciso‐gingival lengths of supra‐gingival tooth structure present. Type 2A.

**Figure 7:**
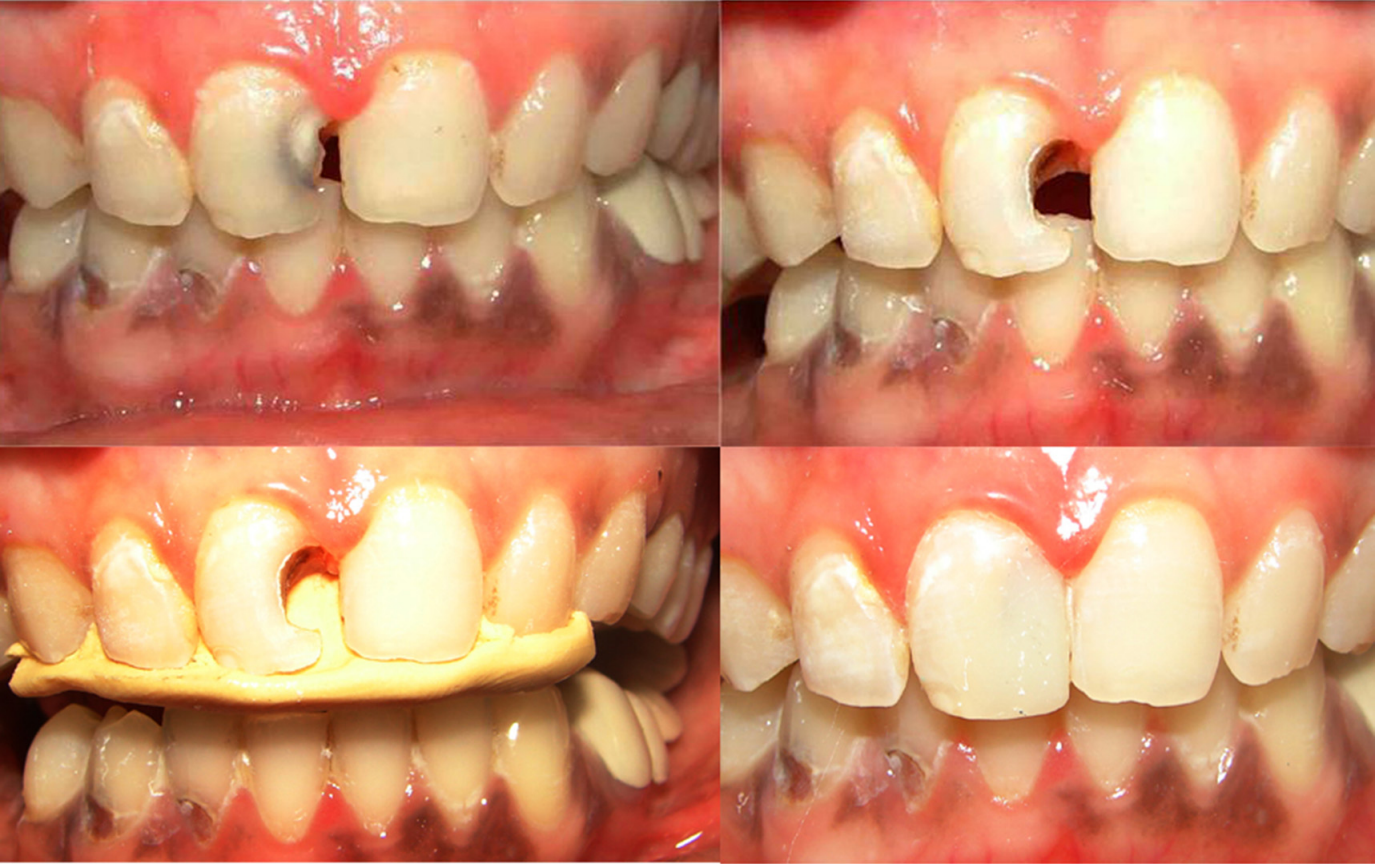
Where defect is 2/3rd or more than 2/3rd the inciso‐gingival lengths of supra‐gingival tooth structure present. Type 2B.

Proximal only defects (G.V. Black Class III):

Type 1: Defects involving either only the labial or palatal surface, with one of the either surface intact (Fig. 5).
Type 2: Through and through defects with restorations being required for both labial and palatal surfaces.
Type 2A: Where defect is less than 2/3rd the inciso‐gingival lengths of supragingival tooth structure present (Fig. 6).
Type 2B: Where defect is 2/3rd or more than 2/3rd the incisogingival lengths of supragingival tooth structure present (Fig. 7).

**Figure 8:**
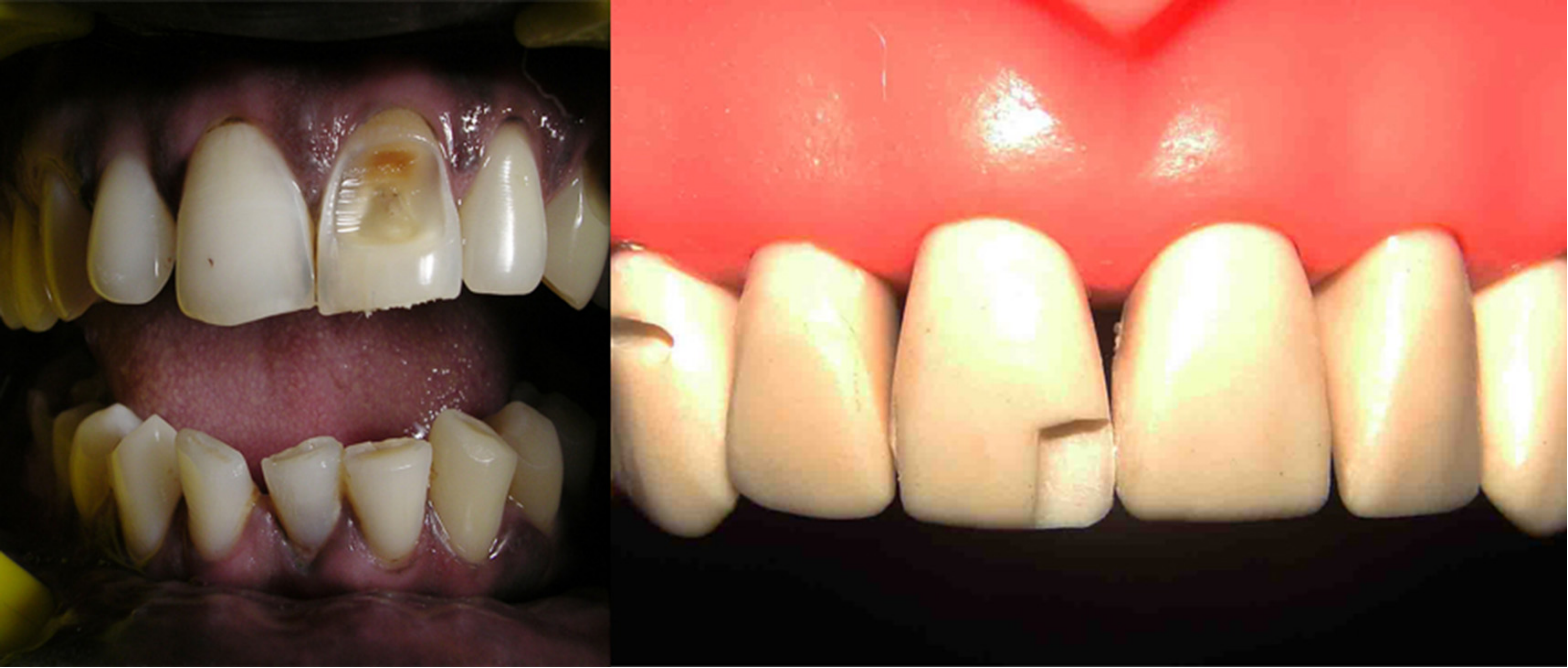
Defects involving either only the labial or palatal surface of incisal and/or proximal regions, with one of the surface intact. Type 3.

**Figure 9:**
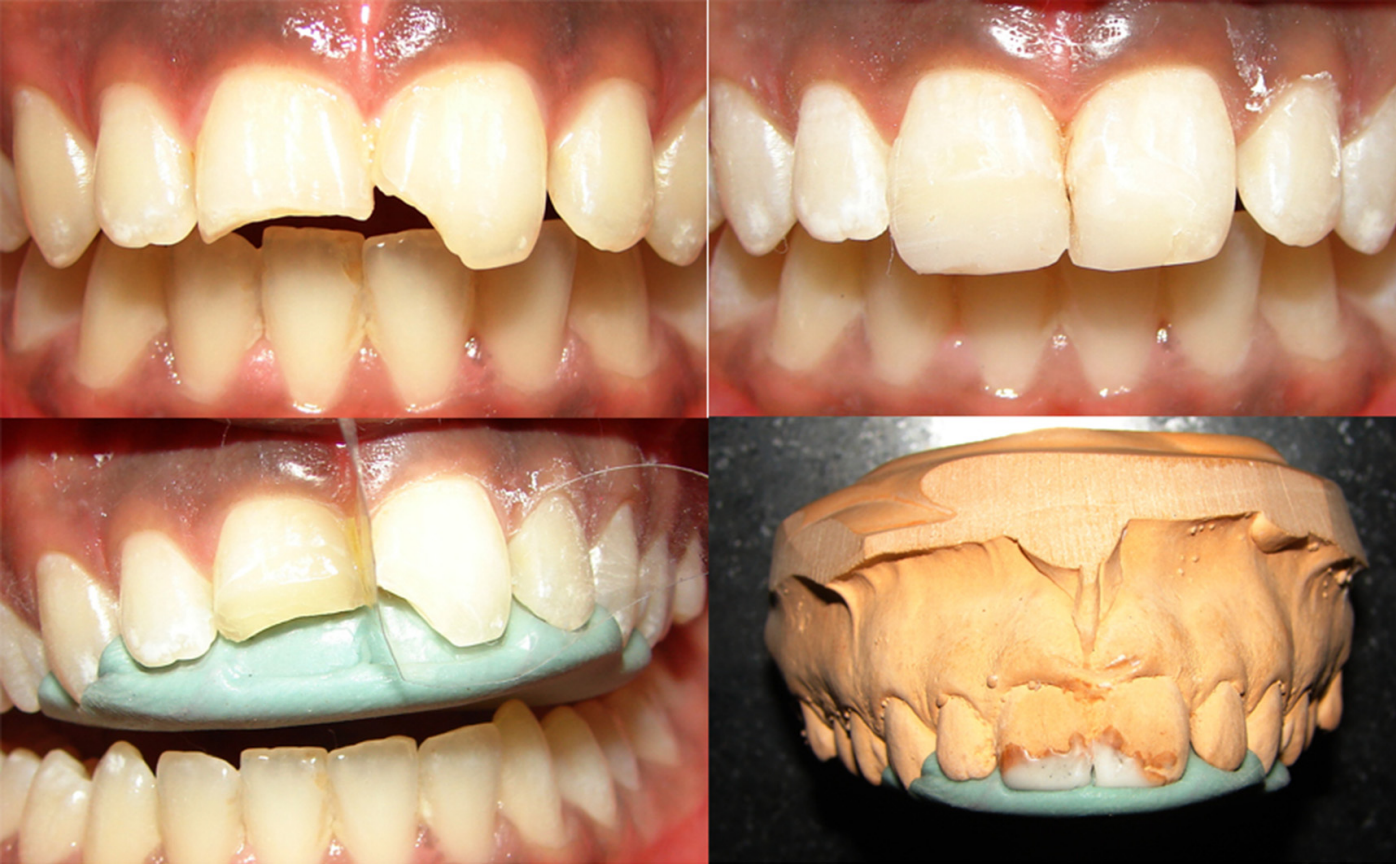
Defects involving both the labial and palatal surfaces of incisal region with or without involvement of proximal regions. Type 4.

**Figure 10:**
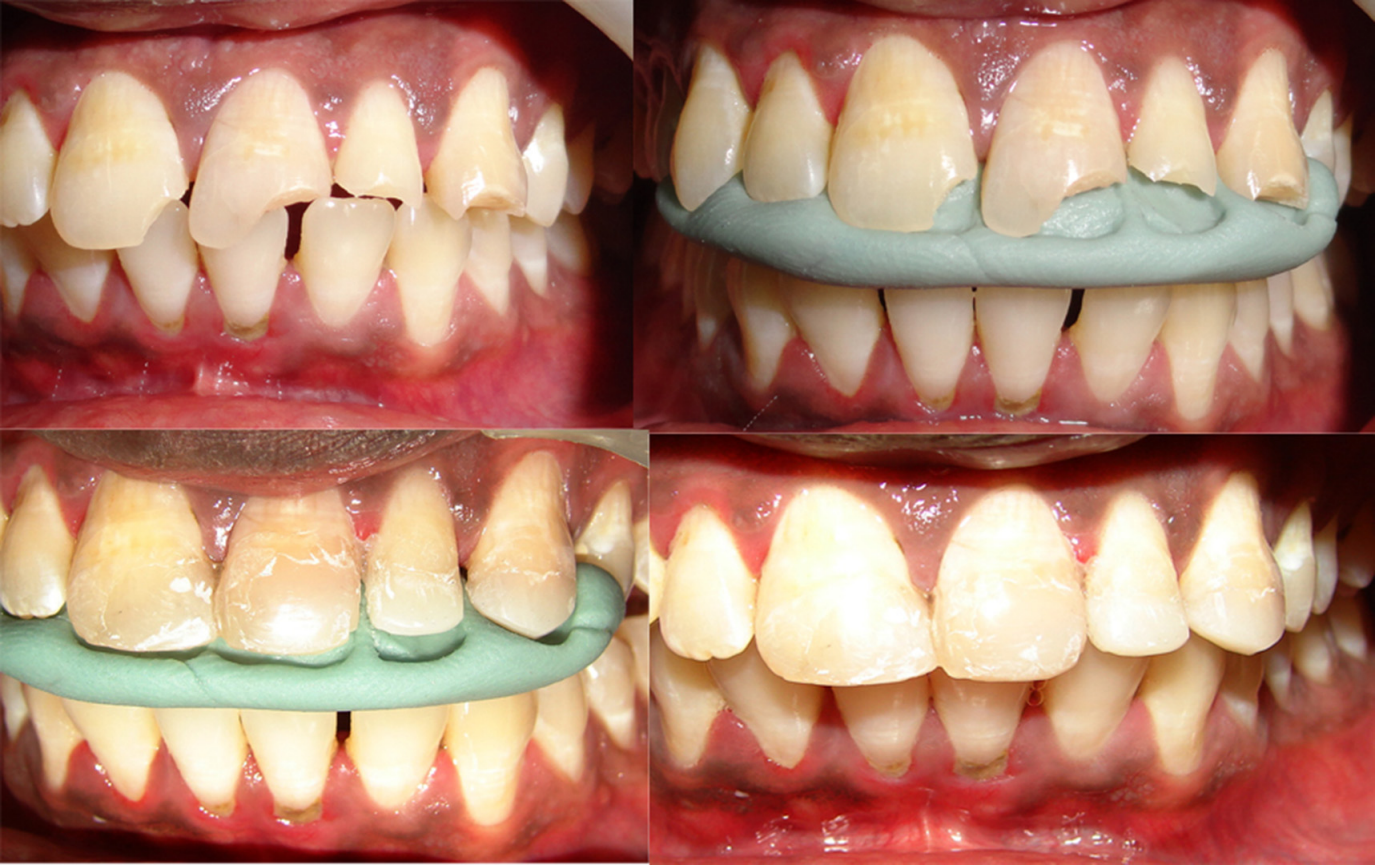
Incisal only defects. Type 4.

Proximoincisal defects /Incisal alone defects (G.V. Black Class IV):

Type 3: Defects involving either only the labial or palatal surface of incisal region with or without involvement of proximal regions, with either of one of the surface intact (Fig. 8).
Type 4: Defects involving both the labial and palatal surfaces of incisal region with or without involvement of proximal regions (Fig. 9). Also incisal only defects (Fig. 10).

## Discussion

Defects which are not through and through like proximal defects Type 1 (Fig. 5) and proximoincisal defects Type 3 defects (Fig. 8) and through and through proximal defects Type 2A (Fig. 6) of smaller dimensions do not require putty index and can be satisfactorily restored free hand with mylar strip alone. Through and through larger defects like proximal defects Type 2B (Fig. 7) and proximoincisal defects Type 4 (Fig. 9) require stiffer putty index with mylar strip technique for satisfactorily restoring the defect with composites both labially and palatally.

Increased incisogingival lengths of the defect require adequate support and rigidity offered by putty index for composite placement, than compared to axial depth dimension of the defect. Also when the axial depth of defect is more than 2/3rd distance toward center of tooth it mostly results in pulp exposure which may require different treatment plan requiring endodontic intervention. In restoring through and through incisal defects stiffer putty index matrix is required for adequate rigid support than flexible mylar strip (Fig. 10). As presented in this case report series satisfactory restoration of anterior teeth with composites in a predictable and acceptable manner can be achieved by diligent usage of putty index matrix with mylar strip especially in situations where the defect to be restored is of greater dimensions. And the proposed classification for selection of the matrix technique for specific clinical situation can be a useful guide for the clinician in restoration of anterior teeth in a better manner.

## Conclusion

The matrix technique described here combines advantages of both flexible and rigid matrix in anterior composite restorations to re‐establish form and contour of tooth structure. Using mylar strip instead of teflon tape provide advantages, (1) utilizing the mylar strip for contouring the labial aspect of composite restoration thereby preventing the gingival overextension of the restoration, and (2) overcomes the problem in adapting the teflon tape around the anterior tooth. The technique and classification proposed in this article is a promising one and can be utilized for varied clinical scenarios both in single tooth or multiple teeth management.

## Authorship

Dr. IAS: (Corresponding Author) contributed to the compilation of cases, writing the manuscripts and conceiving the concept of the technique and classification described. Dr. RM: involved in editing and proof reading of the manuscript and references compilation. Dr KS: involved in editing and language checking of the manuscript. Dr. PB: involved in editing the case photograph. Dr. VVK: provided contribution of his patient works.

## Conflict of Interest

Author denies any conflict of interest.
